# Induction of Urokinase Activity by Retinoic Acid in Two Cell Lines of Neuronal Origin

**DOI:** 10.3390/biomedicines7030070

**Published:** 2019-09-12

**Authors:** Luka Horvat, Josip Madunić, Martina Grubar, Mariastefania Antica, Maja Matulić

**Affiliations:** 1 Department of Molecular Biology, Faculty of Science, University of Zagreb, Horvatovac 102A, 10000 Zagreb, Croatia; luka.horvat@biol.pmf.hr (L.H.); josip.madunic@biol.pmf.hr (J.M.); 2 Division of Molecular Biology, Rudjer Boskovic Institute, Bijenicka 54, 10000 Zagreb, Croatia; antica@irb.hr

**Keywords:** neuroglioblastoma cell line, glioblastoma, retinoic acid, PARP1, urokinase, epithelial-mesenchymal transition

## Abstract

Retinoic acid is one of the most well-known agents able to induce differentiation in several types of tumours. Unfortunately, most of the tumours are refractive to the differentiation cues. The aim of this investigation was to analyse the effects of prolonged treatment with retinoic acid on two cell lines of neural origin refractive to differentiation. Cells were also treated with retinoic acid in combination with a poly(ADP-ribosyl) polymerase (PARP) inhibitor because PARP1 is a known chromatin modulator and can influence the process of differentiation. The main methods comprised tumour cell line culturing and treatment; analysis of RNA and protein expression after cell treatment; as well as analysis of urokinase activity, migration, and proliferation. Both cell lines continued to proliferate under the prolonged treatment and showed increase in urokinase plasminogen activator activity. Analysis of gene expression and cell phenotype revealed different mechanisms, which only in neuroblastoma H4 cells could indicate the process of epithelial-mesenchymal transition. The data collected indicate that the activity of the urokinase plasminogen activator, although belonging to an extracellular protease, does not necessary lead to epithelial-mesenchymal reprogramming and increase in cell migration but can have different outcomes depending on the intracellular milieu.

## 1. Introduction

Among many hallmarks of cancer, the prevailing group of features is considering the inability of tumour cells to stop proliferating, either through avoiding senescence, differentiation, cell cycle arrest, or programmed cell death [1]. Although different chemotherapeutics aim to induce tumour cell death, there are also possibilities to cure tumours by induction of the differentiation process, which diminishes the cell’s ability to proliferate. While the treatment of some forms of promyelocytic leukaemia by derivates of retinoic acid is a standard therapy causing cell differentiation, other types of tumours and leukaemia are mostly refractive to differentiation [2,3,4]. The most common differentiation agent is retinoic acid (RA; all-trans retinoic acid, ATRA), a compound derived from the vitamin A [5]. In the canonical pathway, RA binds to retinoic acid receptors (RAR) and retinoid X receptors (RXR) in the nucleus, which belong to the steroid-thyroid superfamily. The receptors then bind to specific DNA sequences on the target promoters and recruit the transcription machinery [5]. The receptors without bound ligands act as transcription repressors in a complex with chromatin modifiers. RAR was also been found to be able to bind to peroxisome proliferator-activated receptors (PPAR) if certain adaptor molecules are present in the cell and, in this way, can regulate another set of genes [6]. RA can act as a morphogen, and its most known target genes are *HOX* genes [7]. Therefore, it is involved in complex processes of vertebrate neurogenesis, neural stem cell differentiation, and development of several specific types of neurons [5,8]. Pathways involved in RA-mediated embryonic stem cell differentiation toward neuronal tissue involve interactions with different pathways, including Notch, Wnt, MAP kinases, Src kinases, inhibition of GSKβ, and others [7,8].

In this work, we combined the retinoic acid treatment with poly(ADP-ribosyl)polymerase (PARP) inhibition. PARP1 is an enzyme involved in the process of poly(ADP-ribosyl)ation, the addition of polyADP-ribose chains on certain proteins in order to change the chromatin structure and to facilitate the process of DNA damage repair. PARP1 can modify some transcription factors and consequently participate in stem cell establishment and the differentiation process. Due to its influence on the chromatin structure and transcription, we investigated whether it could contribute to retinoic acid-induced cellular processes [9].

In this article, we analysed the changes in two tumour cell lines of neural origin after treatment with retinoic acid and a PARP inhibitor for a prolonged time period. The treated cells continued to proliferate and increased urokinase activity. As this extracellular protease is involved in tissue remodelling and could be involved in epithelial-mesenchymal transition [10,11,12], we analysed the cellular features and the expression of a set of genes involved in these processes.

## 2. Materials and Methods

### 2.1. Cell Culture and Growth Assessment

Glioblastoma (A1235) and neuroglioblastoma (H4) cells were cultured in Dulbecco’s Modified Eagle Medium (DMEM, Sigma-Aldrich, Taufkirchen, Germany) supplemented with 10% foetal bovine serum (Sigma–Aldrich) at 37 °C and 5% CO_2_. A1235 cells were a kind gift from S. A. Aaronson (National Cancer Institute, Bethesda, MD, USA) [13]. Neuroblastoma H4 cells were commercially available at ATCC (Manassas, VA, USA). The cells were tested for the presence of mycoplasma with the EZ-PCR Mycoplasma Test Kit (Biological Industry, Beit-Haemek, Israel).

Cells were treated with ATRA (all-trans retinoic acid) (Sigma-Aldrich) and PJ-34, a PARP inhibitor (Sigma-Aldrich). Control cells were treated with DMSO (Sigma-Aldrich). For prolonged treatment, cells were treated with 10 µM ATRA and 20 µM PJ-34 every second day and reseeded if needed. The 10-µM ATRA treatment for 5–10 days is considered the standard condition for neuroblastoma cell differentiation [14], and 20 µM PJ-34 was shown to be effective in PARP inhibition [15].

Cell growth was assessed by crystal violet staining. Cells were seeded on a 96-well plate in multiplicate, and every day, one set of cells was fixed with cold methanol. At the end of the assay, the plate was stained with crystal violet and, after dissolving in 1% sodium dodecyl sulphate (SDS), absorbance was measured on 595 nm by a microplate reader [16].

### 2.2. Analysis of Enzymatic Activity

Urokinase-type plasminogen activator (uPA) activity was assayed by radial caseinolysis of the conditioned media as described previously [17]. Samples were measured in duplicates and in two biological replicas and compared with the standard uPA (Leo Pharmaceutical Products, Ballerup, Denmark) curve. Experiments were repeated at least two times.

Metalloproteinase activity was examined in the conditioned media by zymography on the polyacrylamide gel copolymerized with gelatine, according to protocol [18].

### 2.3. RNA Preparation, cDNA Synthesis, and Quantitative Real-Time PCR

Total RNA was extracted from cells using TRI Reagent (Sigma), and cDNA was synthesized from 2 µg of total RNA by Primescript RTase (Takara, Kusatsu, Japan) according to manufacturer’s instructions. Quantitative real-time PCR (qRT-PCR) was performed using GoTaq^®^ qPCR Master Mix (Promega, Madison, WI, USA) in a 7500 Fast Real-Time PCR system (Applied Biosystems, ThermoFisher Scientific, Waltham, MA, USA). Gene expression was validated by comparison with *HPRT* gene expression. Primer sequences, designed by IDT PrimerQuest software package (Integrated DNA Technologies, Inc., Coralville, IA, USA) used for PCR reactions, are listed in Supplementary Table S1. Some of the primer sequences, such as those for *HPRT*, *HES1*, *c-MYC*, *TGFβ, SMAD4, SMAD7, SNAIL1, SNAIL2, TWIST1, ZEB1*, and *PARG* were used previously [19,20]. 

### 2.4. SDS PAGE and Western Blot

Total cell extracts were prepared using lysis buffer containing a cocktail of protease inhibitors (Carl Roth, Karlshuhe, Germany), as described previously [21]. Protein expression was analysed by Western blot [21]. The primary antibodies used were PAI1 (Becton Dickenson, Franklin Lakes, NJ, USA), uPA (Cusabio Technology LCC, Houston, TX, USA), and β-actin (Santa Cruz, Dallas, Texas, USA). Densitometric analysis was performed using the ImageJ program (version 1.52a, National Institute of Health, Bethesda, MD, USA).

### 2.5. Migration Assays

Migration assay was performed in Transwell chambers, according to methods reported previously [22]. Two to 4 × 10^4^ cells were seeded in DMEM with or without added ATRA and PJ-34, in a Transwell cell culture chamber with the 8-µm pore size (BRAND, Wertheim, Germany). Cells were allowed to migrate for 18 to 24 h toward DMEM supplemented with 10% serum, with or without added ATRA or PJ-34.

### 2.6. Statistical Analysis

Data were statistically analysed using the software package Microsoft Office and GraphPad Prism 5 (Graph Pad Software. Inc, San Diego, CA, USA). The test used for comparison of the results between the control and the treated cells was one-way ANOVA with Tukey posttest. Significance was set at *p*-value < 0.05.

## 3. Results

### 3.1. The Effect of ATRA and PARP Inhibition on Neuroglioblastoma Cell Growth and Morphology

**Figure 1:**
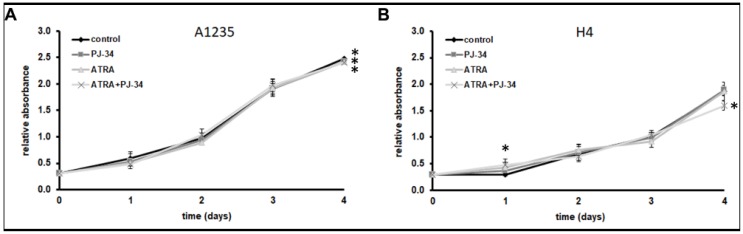
Growth curves of A1235 and H4 cells treated with all-trans retinoic acid (ATRA) and the poly(ADP-ribosyl) polymerase (PARP) inhibitor: Cells were treated every second day with 10 µM ATRA and 20 µM PJ-34 and their combination. Cell proliferation was determined by crystal violet staining and absorbance measurement. (**A**) A1235 cells; (**B**) H4 cells. * The mean values were significantly different from the control (*p* < 0.05).

To investigate whether PARP1 inhibition could influence the cells’ ability to differentiate after ATRA treatment, two tumour cell lines of neural origin, glioblastoma A1235 and neuroglioblastoma H4, were treated with ATRA in combination with a PARP inhibitor, PJ-34. We monitored the cell proliferation under treatment for several days. Both cell lines continued to proliferate at nearly a control rate, except for a little slowing down in growth of A1235 and H4 cells after 4 days of treatment with ATRA and the PARP inhibitor (Figure 1).

**Figure 2:**
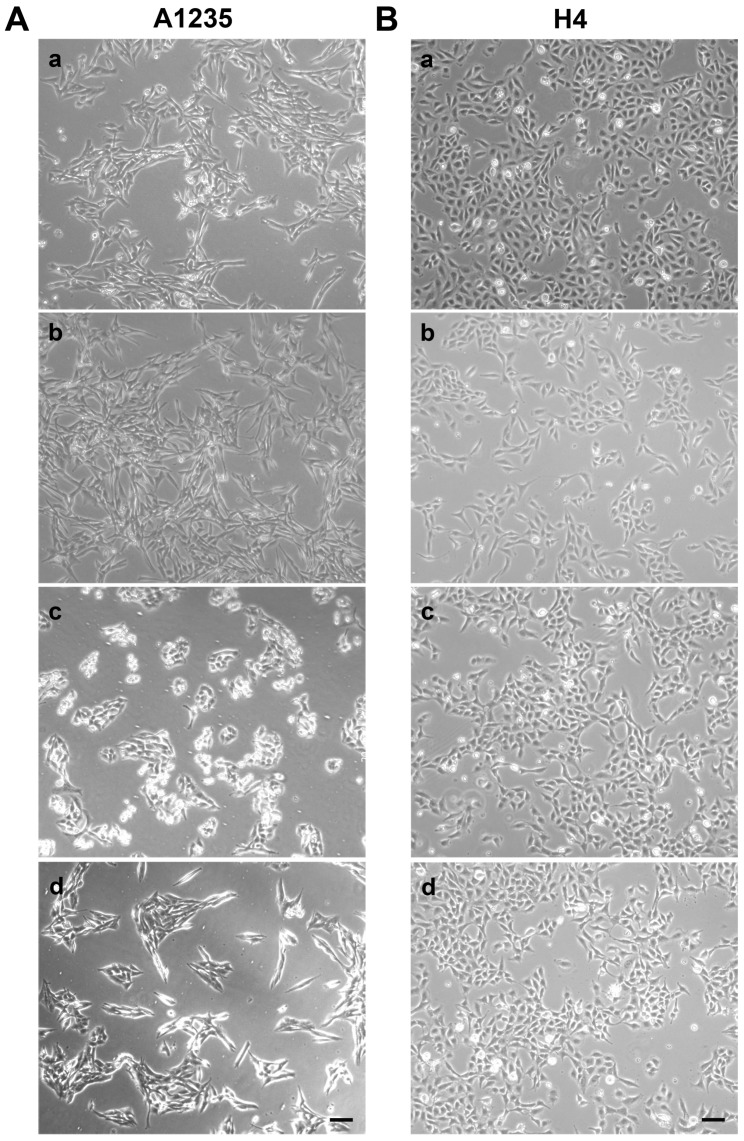
Morphology of A1235 glioblastoma and H4 neuroglioblastoma cells after 9 days of treatment with ATRA and the PARP inhibitor: Microphotographs were taken with epifluorescent microscope Axiovert 40 CFL. (**A**) A1235 cells; (**B**) H4 cells; **a**: control; **b**: cells treated with 20 µM PJ-34; **c**: cells treated with 10 µM ATRA; and **d**: cells treated with 10 µM ATRA and 20 µM PJ-34. Pictures were taken at magnification of 100×, scale bar = 100 µm.

A1235 cells have a fibroblast-like morphology, which showed phenotypic changes during the experiment. Cells treated with ATRA adopted a more epithelial-like shape (Figure 2A). H4 cells have epithelioid morphology, and we did not observe significant changes during the treatment period (Figure 2B).

### 3.2. The Effect of ATRA and PARP Inhibition on Urokinase Activity

**Figure 3:**
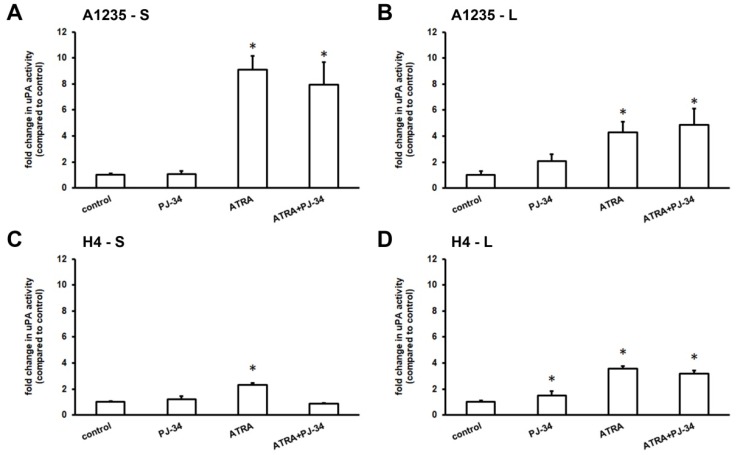
uPA activity of A1235 and cells H4 after treatment with ATRA and the PARP inhibitor: Cells were treated with 10 µM ATRA and 20 µM PJ-34 and their combination every second day. After first day and after more than 9 days of treatment, cells were incubated for 6 h in medium without serum and the urokinase activity was determined in conditioned medium by caseinolysis. The uPA activity was estimated according to the calibration curve of human uPA and protein concentration of corresponding lysates and presented in proportion to control cell values. (**A**) A1235 cells treated for 1 day; (**B**) A1235 cells treated for prolonged time period; (**C**) H4 cells treated for 1 day; and (**D**) H4 cells treated for prolonged time period. S: 1-day treatment; L: prolonged treatment; PJ-34: cells treated with PARP inhibitor; ATRA: cells treated with ATRA; ATRA + PJ-34: cells treated with ATRA and PARP inhibitor. * The mean values were significantly different from the control (*p* < 0.05). Experiments were done two times, and representative results are shown.

Our previous experiments showed that A1235 cells had the ability to increase the activity of urokinase plasminogen activator (uPA) when treated with certain drugs [23,24]. We hypothesized that these cells possibly began the process of epithelial-mesenchymal transition, with changes in the uPA activity as a sign of reprogramming. These experiments led us to test the uPA activity after treatment with ATRA and the PARP inhibitor after one day and after prolonged treatment of up to two weeks. The uPA activity was determined in the serum-free conditioned media by radial caseinolysis. The results revealed that A1235 cells increased the uPA activity several times after ATRA treatment: uPA induction was detected already after one day, as well as after prolonged treatment (Figure 3A,B). When cells grown in ATRA and PJ-34 medium for a longer period were left without a treatment for a week, uPA activity returned to a basal level.

The same experiment was also performed with H4 neuroglioblastoma cells. After one day of treatment, there were only small differences in the uPA activity between the cells treated with ATRA, PJ-34, and their combination and the control, untreated cells (Figure 3C). After more than 9 days of treatment, the uPA activity was increased in cells treated with ATRA. PARP inhibition did not influence this feature (Figure 3D).

### 3.3. The Effect of ATRA and PARP Inhibition on EMT Gene Expression

The uPA system is considered to be involved in EMT, as it can be regulated by mesenchymal master transcription factors [12]. To better understand the processes going on in the cells treated with ATRA, we analysed the expression of several groups of genes in both cell types after treatment with ATRA and the PARP inhibitor. First, we analysed the expression of a set of genes involved in the uPA activity regulation: *uPA*, its inhibitors *PAI1* and *PAI2*, and membrane uPA receptor *uPAR*.

**Figure 4:**
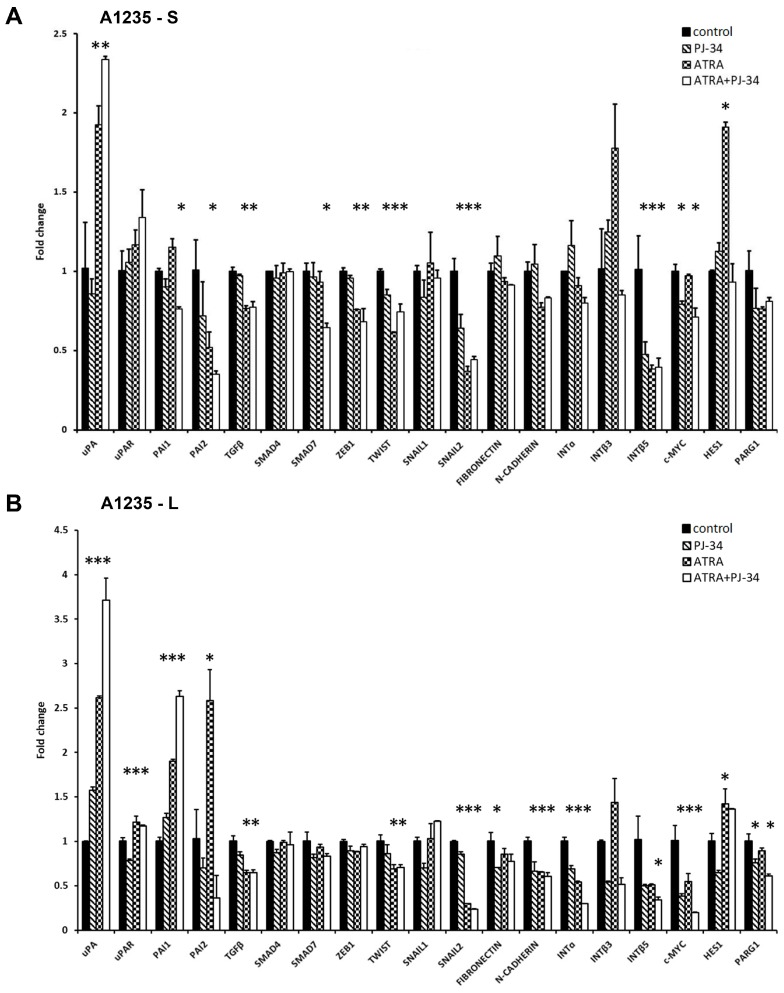

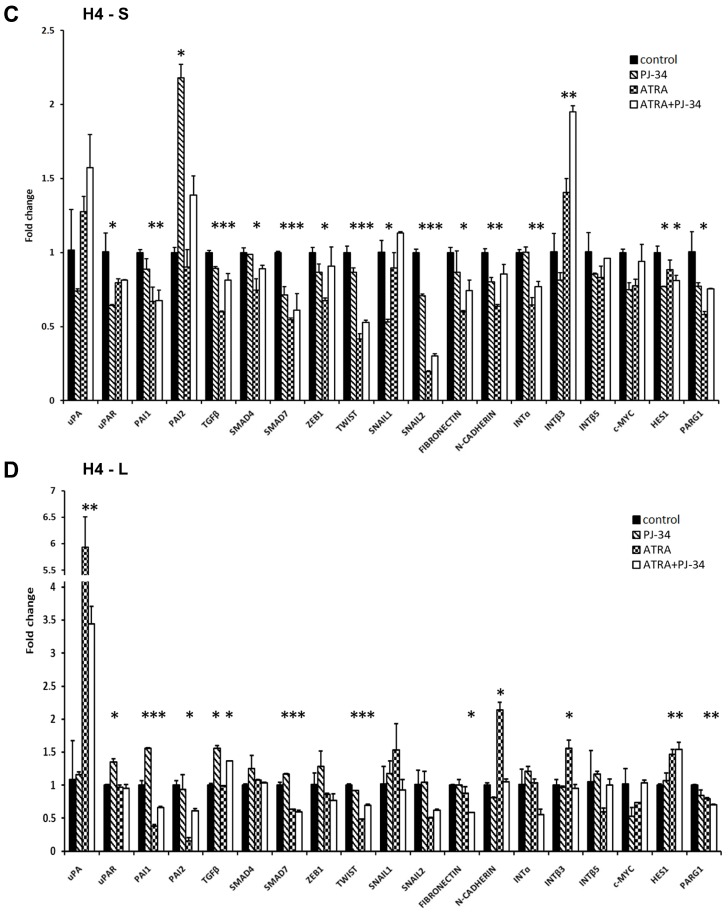
qRT-PCR analysis of the expression of genes involved in the uPA system and EMT in A1235 and H4 cells after 1 and 9 days of ATRA and PJ-34 treatment: Cells were treated with 10 µM ATRA and 20 µM PJ-34 and their combination every second day. RNA was isolated from cells treated for 1 and 9 days, and cDNA was produced and examined by qRT-PCR. Relative gene expression level was obtained by normalization of its values with those of hypoxanthine-guanine phosphoribosyltransferase (*HPRT*) and is presented as a fold change in comparison with untreated control cell values. (**A**) A1235 cells treated for 1 day; (**B**) A1235 cells treated for 9 days; (**C**) H4 cells treated for 1 day; and (**D**) H4 cells treated for 9 days. S: 1-day treatment; L: prolonged treatment; PJ-34: cells treated with PARP inhibitor; ATRA: cells treated with ATRA; ATRA + PJ-34: cells treated with ATRA and the PARP inhibitor. Data are expressed as mean ± SD. * The mean values were significantly different from the control (*p* < 0.05). Experiments with prolonged cell treatment were done at least two times, and representative results are shown.

In the glioblastoma cells, ATRA increased *uPA* expression after one and 9 days. The expression of its inhibitor *PAI1* was also increased after prolonged treatment, while that of *PAI2* was on the very low level. *uPAR* expression showed small changes in the treated cells only after prolonged treatment (Figure 4A,B).

To see whether changes in the uPA system could be linked to the changes in expression of the EMT master transcription factors, we tested *TWIST*, *SNAIL1*, *SNAIL2*, and *ZEB1* [10,11,12]. After one day of treatment with ATRA, a decrease in *ZEB1*, *TWIST*, and *SNAIL2* mRNA was observed, and after 9 days, significant *SNAIL2* and *TWIST* inhibition persisted.

The third group of genes, of which the expression was tested in A1235 cells, were downstream effectors of EMT or its upstream triggers, such as genes involved in the formation of the extracellular matrix (ECM) or cell adhesion [10,11]. *N-CADHERIN* and *INTEGRIN* subunits showed a decrease in expression as a consequence of the prolonged ATRA and PJ-34 treatment. As a TGFβ system can be an initiator of the changes in the EMT, we analysed its expression as well as the expression of downstream *SMAD4* and *SMAD7*, with the latter involved in its negative feedback regulation [11]. In A1235 cells, although *TGFβ* showed small downregulation after ATRA treatment, these changes were not reflected on *SMAD4* and *SMAD7* expression after prolonged treatment. PARP inhibition did not influence *TGFβ* expression. Considering the Notch pathway, its downstream target *HES1* was upregulated by ATRA. We also analysed the expression of *c-MYC*, which showed downregulation after treatment with both ATRA and PJ-34. *PARG1*, an enzyme which cleaves polyADP chains [9], was decreased in dependence on PARP inhibition. 

We also analysed the expression of the same group of genes in H4 neuroblastoma cells treated under the same conditions. Significant changes were observed in the *uPA* and *PAI* expression. *uPA* expression was increased after 9 days of ATRA treatment, and *PAI1* was decreased. *PAI2* expression showed an increase only after 24 h of treatment with the PARP inhibitor, and *uPAR* expression was not significantly changed (Figure 4C,D).

Regarding the expression of the master transcription factors, the expression of *TWIST* and *SNAIL2* was decreased after ATRA treatment. PARP inhibition alleviated ATRA effects on the expression of certain target genes, some master transcription factors, and their downstream targets (Supplementary Table S2). Considering integrins, only *INTEGRIN β3* showed an increase in ATRA and PJ-34 treated cells. After 9 days, the expression of most of the *INTEGRIN* subunits was on the control level. Analysis of TGFβ system expression revealed changes in *TGFβ* and *SMAD7* expression. After 9 days of treatment, *TGFβ* expression showed an increase in samples where the PARP inhibitor was added, while *SMAD7* was decreased in ATRA-treated cells. *HES1* and *PARG1* expression was also changed in ATRA-treated cells (Figure 4C,D).

### 3.4. The Effect of ATRA and PARP Inhibition on the Plasminogen Activation System Proteins

**Figure 5:**
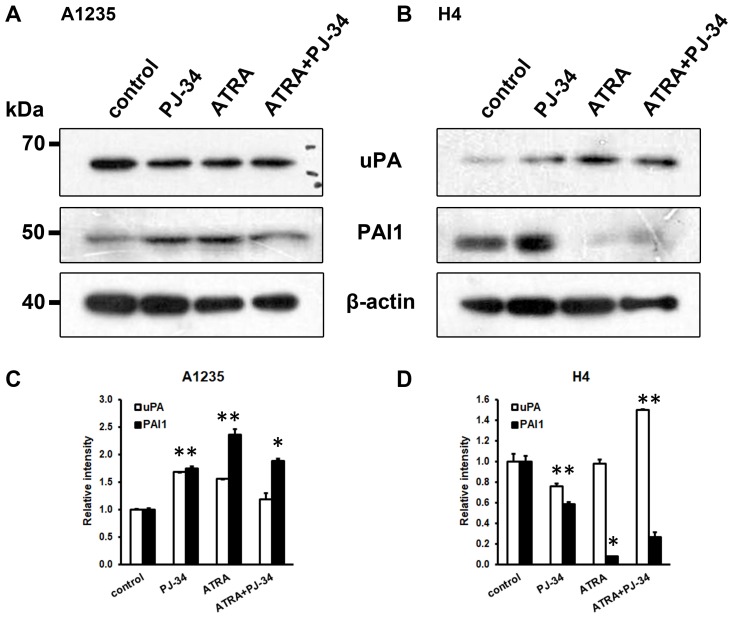
Western blot analysis of uPA and PAI-1 in A1235 and H4 cells after treatment with ATRA and PJ-34: After the prolonged treatment with 10 µM ATRA and 20 µM PJ-34 and their combination, the whole cell lysates were immunoblotted against indicated antibodies. (**A**) Analysis of A1235 cells; (**B**) analysis of H4 cells; and (**C**,**D**) densitometric analysis of western blot films of A1235 and H4 cells, respectively. PJ-34: cells treated with PARP inhibitor; ATRA: cells treated with ATRA; ATRA + PJ-34: cells treated with ATRA and the PARP inhibitor. Data are expressed as mean ± SD. * The mean values were significantly different from the control (*p* < 0.05). Experiments were done two times, and representative results are presented.

The uPA system was also analysed on the protein level. Proteins were isolated from the cells treated for a prolonged time with ATRA and the PARP inhibitor. In A1235 cells, although a small increase in uPA could be observed in some treated cells, there was also an increase in PAI1 in all treated samples (Figure 5A,C). In H4 cells, increase in uPA was detected in cells treated with ATRA in combination with the PARP inhibitor and decrease in PAI1 was detected in all treated cells (Figure 5B,D).

### 3.5. The Effect of ATRA and PARP Inhibition on Cell Migration and Matrix Metalloprotease Expression

**Figure 6:**
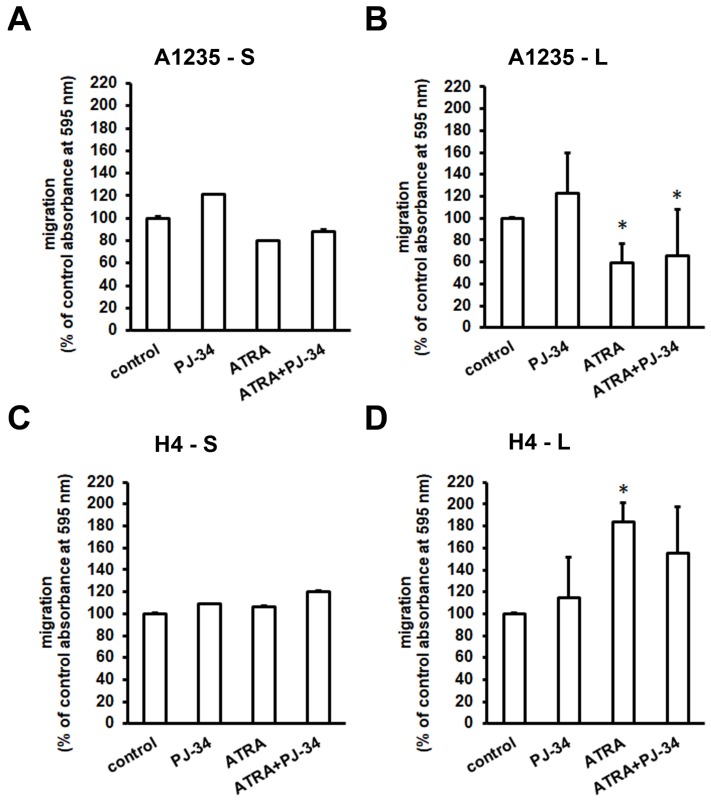
Analysis of cell migration after ATRA and PJ-34 treatment: Cells were seeded in Transwell chambers and allowed to migrate toward the medium with serum in the presence or in the absence of ATRA or PJ-34. Membranes were stained with crystal violet, and the absorbance was measured spectrophotometrically. (**A**) A1235 cells’ migration after 1 day of treatment; (**B**) A1235 cells’ migration after prolonged treatment; (**C**) H4 cells’ migration after 1 day of treatment; and (**D**) H4 cells’ migration after prolonged treatment. S: 1-day treatment; L: prolonged treatment; PJ-34: cells treated with 20 µM PJ-34; ATRA: cells treated with 10 µM ATRA; ATRA + PJ-34: cells treated with 20 µM PJ-34 and 10 µM ATRA inhibitor. * The mean values were significantly different from the control (*p* < 0.05). Experiments were done two times.

In order to examine the effect of urokinase activity and EMT master transcription factor expression on the ability of cells to migrate, we performed cell migration analysis. Cells were seeded in Transwells and exposed to the PARP inhibitor and/or ATRA for ~20 h. After one day of treatment, migration of both A1235 and H4 cells, control and treated, was similar (Figure 6A,C). After prolonged treatment, we observed a decrease in A1235 and an increase in H4 migration after treatment with ATRA (Figure 6B,D).

**Figure 7:**
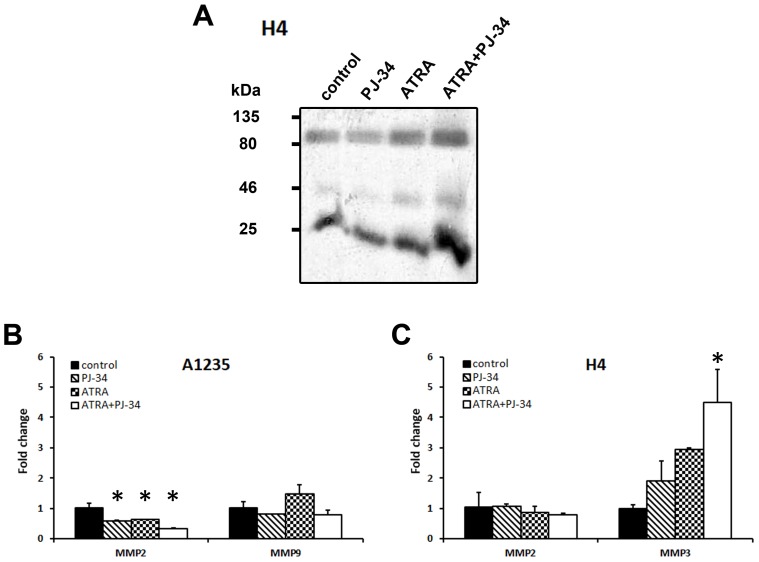
Metalloprotease activity and expression in A1235 and H4 cells after ATRA and PJ-34 treatment: (**A**) Zymography of H4 cells. After the prolonged treatment with 10 µM ATRA and 20 µM PJ-34 and their combination, conditioned H4 cells were collected, concentrated, and analysed by zymography on a gelatine-containing polyacrylamide gel. (**B**) qPCR analysis of MMP expression in A1235 cells; (**C**) qPCR analysis of MMP expression in H4 cells. RNA was isolated from cells after prolonged treatment, cDNA was produced and examined by qRT-PCR. Relative gene expression level was obtained by normalization of its values with those of hypoxanthine-guanine phosphoribosyltransferase (*HPRT*) and is presented as a fold change in comparison with untreated control cell values. PJ-34: cells treated with 20 µM PJ-34; ATRA: cells treated with 10 µM ATRA; ATRA + PJ-34: cells treated with 20 µM PJ-34 and 10 µM ATRA inhibitor; MMP2: metalloproteinase 2; MMP3: metalloproteinase 3; MMP9: metalloproteinase 9. * The mean values were significantly different from the control (*p* < 0.05).

As matrix metalloproteases (MMP) are often involved in the process of migration and are expressed in mesenchymal cells, we also analysed their expression and activity in both cell lines after the prolonged treatment with ATRA and the PARP inhibitor. As several metalloproteases act as gelatinases, their activity was examined by zymography on a gelatine-containing gel [25]. Gelatinases detected in A1235 cells had very weak activity. In H4 cells, there was an increase in gelatinase activity in media obtained from samples treated with ATRA alone and in combination with PJ-34 (Figure 7A and Figure S1). As the technique applied can detect several MMPs, we analysed the expression of MMP2, MMP3, and MMP9 on the RNA level by qPCR. In A1235 cells, only MMP2 and MMP9 were detected and a decrease in MMP2 expression was observed. In H4 cells, only MMP2 and MMP3 were expressed and MMP3 expression was increased after ATRA treatment in combination with PJ-34 (Figure 7B,C).

## 4. Discussion

ATRA is often used as a differentiation agent in certain types of tumour cells and could be considered a targeted chemotherapeutic, with optimal effects. Unfortunately, the number of tumours prone to differentiation is very low, due probably to active signalling pathways forcing cells on proliferation and dominating over differentiations cues [4,26,27]. Ostensive proliferation after ATRA and PARP inhibitor treatment in the neuroblastoma and glioblastoma cell lines investigated indicated insensitivity to drug treatment and cells’ inability to differentiate. Also, there were no neurite outgrowth and significant morphological changes indicating differentiation. Despite this, ATRA did cause changes in intracellular signalling, indicating cellular reprogramming. We first detected an increase in the urokinase activity after RA treatment. Urokinase plasminogen activator is an extracellular enzyme often considered to influence cell migration and invasion and was found to be involved in epithelial-mesenchymal transition (EMT) [12,28]. In this process, by obtaining mesenchymal features, epithelial cells (or cells of other types in the similar process) acquire the ability to migrate, to produce extracellular matrix proteins and proteases, to change their integrin and receptor expression, and to loosen tight and adhesive junctions with neighbouring cells. This reprogramming is a normal process during development but also present in wound healing and other physiological processes [10,11,29]. Nowadays, it comes into the focus of investigation because of its role in tumour spreading and tumour stem cell biology [30,31,32]. Thus, we analysed a set of cellular features involved in this process. 

RA treatment increased urokinase activity in both cell lines of neural origin, but the pattern of induction was not the same. In H4 neuroblastoma cells, we observed minimal changes after 1 day of treatment but the activity significantly increased after prolonged treatment. These changes were reversible, and activity decreased after culturing the cells without the drug. In these cells, increase in urokinase activity could be ascribed to coordinated increase in uPA and decrease in PAI1 expression. In parallel, there was also an increase in metalloprotease activity as well as in cell migration. On the other hand, there were no changes in the epithelial phenotype of H4 cells. Expression of several genes regulating cell attachment, such as *INTEGRINS*, *FIBRONECTIN*, and *N-CADHERIN*, was only sporadically changed, i.e., *N-CADHERIN* was increased only in cells treated with ATRA alone. EMT master transcription factors *TWIST* and *SNAIL2* were downregulated by RA [10,31]. Analysis of TGFβ signalling in the RA-treated cells showed a decrease in *SMAD7* expression, its negative regulator. *TGFβ* expression, on the contrary, showed increase in cells treated with the PARP inhibitor. The PARP inhibitor’s influence on ATRA-dependent expression could be seen in several TGFβ-dependent genes, such as *N-CADHERIN, INTEGRIN*, and *TWIST* [11]. It is known that PARP can regulate physiological responses to TGFβ by PARPylation of the activated Smad proteins. This modification was found to increase Smad complex dissociation from DNA and to decrease gene response to TGFβ signalling and epithelial-mesenchymal transition dependent on it [33,34]. Thus, PARP inhibition could change the duration of TGFβ signals and interfere with signalling induced by ATRA. However, the final outcome seemed to be dependent on the intracellular milieu which determines downstream TGFβ targets and general TGFβ response. Complex relations between TGFβ-induced EMT and PARP activity were also confirmed recently in mammary epithelial cells, which showed inhibition of EMT under PARP inhibition [35]. Therefore, although *uPA*, *PAI1*, and *PAI2* are TGFβ-responsive genes and final changes in the uPA activity and other EMT-like features in H4 cells correlated only with ATRA treatment, H4 cells did not exhibit all of the EMT-like features but it could be ascribed to partial EMT or similar process found in both physiological processes and in tumour biology [29,36].

*c-MYC* expression often correlates with cell proliferation ability, and in H4 cells, it was not significantly decreased after prolonged treatment. Duffy et al. [27] analysed neuroblastoma cells with *N-MYC* overexpression during ATRA treatment and concluded that N-Myc regulated differentiation-relevant genes in the opposite way in comparison with ATRA. They found N-Myc-related inhibition of TGFβ signalling and interactions of their networks on multiple levels. The authors concluded that N-Myc expression dominated over other signalling pathways and disabled differentiation network activation by ATRA. Chen et al. [37] found that the defective regulation of *c-MYC* or its upstream signalling in breast tumours leads to *c-MYC* unresponsiveness to TGFβ signals. H4 cells could have similar mechanisms of differentiation avoidance, possibly not dependent directly on *c-MYC* but on proliferation signalling cooperating with Myc, such as Akt pathways [38]. 

The A1235 glioblastoma cell line shared with H4 cells the ability to avoid differentiation and to continue to proliferate under RA treatment, as well as to increase urokinase activity. However, in these cells, the urokinase activity showed different dynamics; it showed immediate upregulation which remained on the high level during treatment. As in H4 cells, the activity was downregulated after RA removal. Increase in the urokinase activity was accompanied by the upregulation in *uPA* expression and also by the upregulation of its inhibitor *PAI1*. *PAI2* expression was low, so we could suppose that it did not influence significantly the uPA activity. On the protein level, there was no significant increase in the uPA expression correlating with activity. On the contrary, increase in PAI1 expression was detected in the cell lysates. A1235 cells were described previously to increase the urokinase activity when treated with chemotherapeutics, but it was shown that ZEB1 could be involved in coordinated increases in uPA and decreases in PAI1 expression [12]. RA regulated urokinase activity differently. Our hypotheses are that extracellular uPA activity could be regulated on the level of uPA or PAI1 secretion or by the differential expression of some other specific inhibitors, which were found recently [39,40,41,42].

Despite the increase in the urokinase activity, a number of changes observed in RA-treated A1235 cells indicated the absence of EMT-like reprogramming. Cells showed the decrease in migration ability, low expression of metalloproteases, and epithelioid-like morphology. Also, there was a decrease in *TWIST* and *SNAIL2* expression, as well as in those of integrins and *N-CADHERIN.* The TGFβ system in A1235 cells correlated with ATRA treatment, causing the downregulation of *TGFβ* expression. 

ATRA treatment was found to cause the reversal of EMT in several experimental systems. Cui et al. [43] analysed its effects on hepatocellular carcinoma cells and found the suppression of proliferation, migration, and invasion, as well as downregulation of the mesenchymal genes *TWIST*, *SNAIL*, *N-CADHERIN*, and *VIMENTIN*. Zanetti et al. [44] also found that ATRA induces epithelial differentiation program in breast tumour cells. ATRA was also found to increase the amounts of VE-cadherin, β-catenin, as well as cell-to-cell contacts and to decrease the cell migration. SNAIL1 was decreased when ATRA was applied in combination with EGF. In addition, it was found that ATRA activated the TGFβ pathway which possibly did a functional switch to antimigratory behaviour. 

We could conclude that both ATRA-dependent pathways and PARP inhibition induce changes in cell-specific signalling network, dependent on the intracellular milieu. Although both cell lines of neural origin increased the activity of urokinase after ATRA treatment, H4 cells showed also some of the hallmarks of EMT, while glioblastoma A1235 cells showed signs of the opposite process.

## 5. Conclusions

Glioblastoma A1235 and neuroglioblastoma H4 cells were resistant to the induction of differentiation by ATRA and PARP inhibition. Both cell lines increased uPA activity after ATRA treatment, but the regulation of uPA system elements as well as changes in gene expression network were cell-type specific. While ATRA induced changes resembling the hallmarks of EMT in one cell line, in the other, it caused the features of the opposite process. PARP inhibition did not have a consistent effect by itself or in addition to ATRA. The data indicate that the activity of urokinase plasminogen activator, although an extracellular protease, does not necessarily lead to epithelial-mesenchymal reprogramming but could have different outcomes dependent on the intracellular milieu.

## Supplementary Materials

The following are available online at https://www.mdpi.com/2227-9059/7/3/70/s1.
