# Supplementary material for: Induction of Urokinase Activity by Retinoic Acid in Two Cell Lines of Neuronal Origin
Source: Biomedicines. 2019 Sep 12;7(3):70. doi: 10.3390/biomedicines7030070 (PMC6784121; doi:10.3390/biomedicines7030070)
Supplement: Supplementary file 1 [file biomedicines-07-00070-s001.pdf]

Supplementary data

Table S1. List of primers used for qRT-PCR

HPRT	F: 5'-CTTTGCTGACCTGCTGGATT-3'<br>R: 5’-TCCCCTGTTGACTGGTCATT-3'
HES1	F: 5’-AGCACAGAAAGTCATCAAAGC-3'<br>R: 5’-CCGCGAGCTATCTTTCTTCA-3'
c-MYC	F: 5’-CTGCTTAGACGCTGGATTT-3'<br>R: 5’-CTCCTCGTCGCAGTAGAAA-3'
PAI1	F: 5’-CTGGTGAATGCCCTCTACTTC-3’<br>R: 5’-TGCTGCCGTCTGATTTGT-3’
PAI2	F: 5’-GCAGATCCAGAAGGGTAGTTATC-3’<br>R: 5’-TTGATTGCAGAGCTGAGAGAG-3’
SMAD4	F: 5’-CGCGGTCTTTGTACAGAGTTA-3’<br>R: 5’-GATGACACTGACGCAAATCAAA-3’
SMAD7	F: 5’-ACCCGATGGATTTTCTCAAACC-3’<br>R: 5’-GCCAGATAATTCGTTCCCCCT-3’
SNAIL1	F: 5’-TCTAGGCCCTGGCTGCTACA-3’<br>R: 5’-CATCTGAGTGGGTCTGGAGGT-3’
SNAIL2	F: 5’-CCTGGTCAAGAAGCATTTCAAC-3’<br>R: 5’-GAGGATCTCTGGTTGTGGTATG-3’
TGFβ	F: 5’-GACACCAACTATTGCTTCAG’-3’<br>R: 5’-AGAAGTTGGCATGGTAGCCC-3’
TWIST1	F: 5'-CGGAGACCTAGATGTCATTGTTT-3'<br>R: 5'-ACGCCTGTTTCTTTGAATTTG-3'
uPA	F: 5’-GGAGATGAAGTTTGAGGTGGAA-3’<br>R: 5’-CTCCTTGGAACGGATCTTCAG-3’
uPAR	F: 5’-TTGAAGATCACCAGCCTTACC-3’<br>R: 5’-GGTAACGGCTTCGGGAATAG-3’
ZEB1	F: 5’-GGCAGATGAAGCAGGATGTA-3’<br>R: 5’-GACAGCAGTGTCTTGTTGTTG-3’
FIBRONECTIN	F: 5'-GCCAGTCCTACAACCAGTATTC-3'<br>R: 5'- CTTCTCTGTCAGCCTGTACATC-3'
INTEGRIN αV	F: 5'- GTTGGGAGATTAGACAGAGGAAAG-3'<br>R: 5'- GCAGACGACTTCAGAGAATAGG-3'
INTEGRIN β5	F: 5'-GCAAGATCTATGGCCTTTCT-3'<br>R: 5'-TGTCGAGCAGTTACAGTTGTC-3
INTEGRIN β3	F: 5'-GTTACTGCCGTGACGAGATT-3'<br>R: 5'-CGACACAGTCATCCTCATTCTT-3'
CADHERIN N	F: 5'-GCCAAGCCAGGACAGATAAT-3'<br>R: 5'-GCAGCCTCAGGTGATAATCTAA-3'
PARG	F: 5'-TCCAGAATGGGAAAGATGTG-3'<br>R: 5'-CTCAGCATAGCCTGTGTATTC-3'
MMP2	F: 5'-CGTGGTGAGATCTTCTTCTTC-3'<br>R: 5'-CCTCGTATACCGCATCAATC-3'
MMP3	F: 5'-GCCAGGGATTAATGGAGATG-3'

	R: 5'-GTGTTGGCTGAGTGAAAGAG-3'
MMP9	F: 5'-TTGACAGCGACAAGAAGTG-3'<br>R: 5'-GGCACTGAGGAATGATCTAAG-3'

Table S2.

A. Changes in expression of the genes involved in the uPA system and EMT in A1235 cells after 1 day of treatment with ATRA and PJ-34

Gene	C : PJ	C: ATRA	C : ATRA+PJ	ATRA : ATRA+PJ
uPA		↑	↑↑	
uPAR				
PAI1			↓	↓↓
PAI2			↓	
TGFβ		↓↓	↓↓	
SMAD4				
SMAD7			↓	↓
ZEB1		↓	↓↓	
TWIST	↓	↓↓	↓↓	↑
SNAIL1				
SNAIL2	↓	↓↓	↓↓	
FIBRONECTIN				
N-CADHERIN				
INTα				
INTβ3				↓
INTβ5	↓	↓	↓	
c-MYC	↓		↓↓	↓↓
HES1		↑↑		↓↓
PARG1				

B. Changes in expression of the genes involved in the uPA system and EMT in A1235 cells after prolonged treatment with ATRA and PJ-34

Gene	C : PJ	C: ATRA	C : ATRA+PJ	ATRA : ATRA+PJ
uPA	↑	↑↑	↑↑	↑↑
uPAR	↓	↑	↑	
PAI1	↑	↑↑	↑↑	↑↑
PAI2		↑		↓↓
TGFβ		↓↓	↓↓	
SMAD4				
SMAD7				
ZEB1				
TWIST		↓↓	↓↓	
SNAIL1				
SNAIL2	↓	↓↓	↓↓	↓

FIBRONECTIN	↓			
N-CADHERIN	↓	↓	↓↓	
INTα	↓↓	↓↓	↓↓	↓↓
INTβ3				↓↓
INTβ5			↓	
c-MYC	↓	↓	↓↓	
HES1		↑		
PARG1	↓		↓↓	↓

C. Changes in expression of the genes involved in the uPA system and EMT in H4 cells after 1 day of treatment with ATRA and PJ-34

Gene	C : PJ	C: ATRA	C : ATRA+PJ	ATRA : ATRA+PJ
uPA				
uPAR	↓			
PAI1		↓	↓	
PAI2	↑↑			↑
TGFβ	↓	↓↓	↓↓	↑↑
SMAD4		↓		
SMAD7	↓	↓↓	↓	
ZEB1		↓		
TWIST	↓	↓↓	↓↓	
SNAIL1	↓↓			
SNAIL2	↓↓	↓↓	↓↓	↑↑
FIBRONECTIN		↓		
N-CADHERIN	↓	↓↓		↑
INTα		↓↓	↓	
INTβ3		↑	↑↑	↑
INTβ5				
c-MYC				
HES1	↓		↓	
PARG1		↓		

D. Changes in expression of the genes involved in the uPA system and EMT in H4 cells after prolonged treatment with ATRA and PJ-34

Gene	C : PJ	C: ATRA	C : ATRA+PJ	ATRA : ATRA+PJ
uPA		↑↑	↑	↓
uPAR	↑↑			
PAI1	↑↑	↓↓	↓↓	↑↑
PAI2		↓↓		
TGFβ	↑↑		↑↑	↑↑
SMAD4				
SMAD7	↑↑	↓↓	↓↓	
ZEB1				

TWIST	↓↓	↓↓	↓↓	↑↑
SNAIL1				
SNAIL2				
FIBRONECTIN			↓	↓
N-CADHERIN		↑↑		↓↓
INTα				
INTβ3		↑↑		↓↓
INTβ5				
c-MYC				
HES1		↑	↑	
PARG1		↓	↓↓	

C : PJ: statistical comparison of relative mRNA expression in control cells and cells treated with 20 µM PJ-34; C : ATRA: statistical comparison of relative mRNA expression in control cells and cells treated with 10 µM ATRA; C : ATRA+PJ statistical comparison of relative mRNA expression in control cells and cells treated with 10 µM ATRA and 20 µM PJ-34; ATRA : ATRA + PJ: statistical comparison of relative mRNA expression in cells treated with ATRA and those treated with 10 µM ATRA and 20 µM PJ-34; ↓: downregulation; ↑: upregulation; one arrow: p-value < 0.05; two arrows: p-value < 0.01. Relative expression was determined by qPCR. Statistical analysis was done by ANOVA and Tukey test.

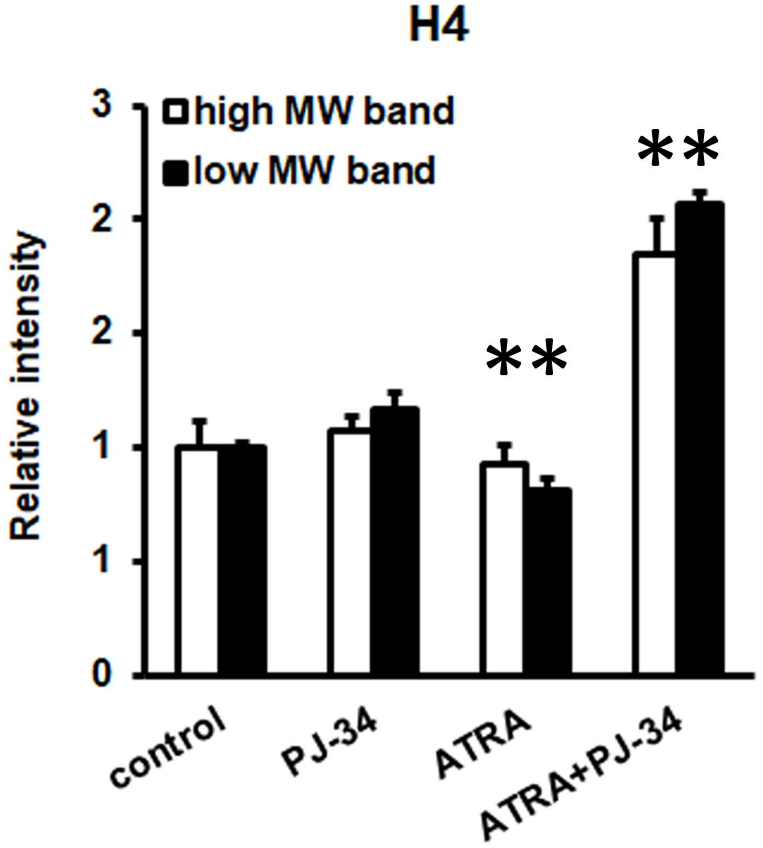

Figure S1. Densitometric analysis of metaloproteinase activity of H4 cells on a gelatine gel. After the prolonged treatment with 10 µM ATRA and 20 µM PJ-34 and their combination, conditioned media from H4 cells were collected, concentrated and analysed by zymography on a gelatine-containing polyacrylamide gel. Densitometric analysis of high and low molecular weight bands was done (Fig. 7. A). PJ-34: cells treated with 20 µM PJ-34; ATRA: cells treated with 10 µM ATRA; ATRA+PJ-34: cells treated with 20 µM PJ-34 and 10 µM ATRA inhibitor. * the mean values were significantly different from control ($p < 0.05$).
